# An approach to quantify *ortho*-phthalaldehyde contamination on work surfaces

**DOI:** 10.1093/annweh/wxad039

**Published:** 2023-07-12

**Authors:** Caitlyn A Rogers, Sharyn E Gaskin, Leigh D Thredgold, Tara L Pukala

**Affiliations:** Adelaide Exposure Science and Health, School of Public Health, University of Adelaide, Adelaide, South Australia, 5005, Australia; Adelaide Exposure Science and Health, School of Public Health, University of Adelaide, Adelaide, South Australia, 5005, Australia; Adelaide Exposure Science and Health, School of Public Health, University of Adelaide, Adelaide, South Australia, 5005, Australia; Department of Chemistry, School of Physical Sciences, University of Adelaide, Adelaide, South Australia, 5005, Australia

**Keywords:** *o*-phthalaldehyde, surface sampling, Threshold Limit Value– Surface Limit (TLV– SL)

## Abstract

*Ortho*-phthalaldehyde (OPA) is used as a high-level disinfectant for reusable medical devices in healthcare settings. The ACGIH recently adopted a Threshold Limit Value–Surface Limit (TLV–SL; 25 µg/100 cm^2^) for OPA surface contamination to prevent induction of dermal and respiratory sensitization following dermal exposure. However, there is no current validated method to measure OPA surface contamination. This study aimed to develop a standardized approach for sample collection and quantitative determination of OPA from work surfaces for use in risk assessment practices. The reported method utilises readily available commercial wipes to collect surface samples coupled with direct detection of OPA via liquid chromatography time of flight mass spectrometry (LC–ToF–MS). This approach avoided complex derivatization steps commonly required for the analysis of aldehydes. Method evaluation was conducted in accordance with the Occupational Safety and Health Administration (OSHA) surface sampling guidelines. Overall recoveries of 25 µg/100 cm^2^ of OPA from stainless steel and glass surfaces were 70% and 72%, respectively. The reported LOD for this method was 1.1 µg/sample and the LOQ was 3.7 µg/sample. OPA remained stable on the sampling medium for up to 10 days, when stored at 4 °C. The method was demonstrated in a workplace surface assessment at a local hospital sterilising unit, successfully detecting OPA on work surfaces. This method is intended to supplement airborne exposure assessment and provide a quantitative assessment tool for potential dermal exposure. When used in conjunction with a thorough occupational hygiene program that includes hazard communication, engineering controls, and personal protective equipment, skin exposure and consequent sensitization risks in the workplace can be minimized.

**What’s important about this paper?**

This study demonstrated a surface sampling and analytical method for the measurement of *ortho*-phthalaldehyde (OPA) on surfaces. OPA is increasingly used in healthcare settings for disinfection but may induce dermal and respiratory sensitization. The developed sampling and analytical method can be used as part of a risk assessment to evaluate the potential for dermal exposures to OPA.

## Introduction

*Ortho*-phthalaldehyde (OPA) is increasingly being used as an alternative to glutaraldehyde in healthcare settings as an effective disinfectant for heat-sensitive medical instruments (Tucker 2014). OPA is a dialdehyde that exhibits similar reactivity and effectiveness as a biocide but has a lower vapour pressure (0.69 Pa) than glutaraldehyde (80 Pa), therefore, it has been marketed as a ‘safer’ alternative (Chen et al. 2015). Marketing literature, local anecdotal information and a Canadian survey in British Columbian hospitals (Rideout et al. 2005) indicate that the use of OPA is widespread and on the rise. The US National Institute of Health (National Institute of Health 2007) estimated that more than 300 000 healthcare workers are exposed to OPA. Typical concentration of the active ingredient is 0.55% to 0.60% (product Cidex^TM^ OPA) at which a wide spectrum of antimicrobial activity and high level of disinfection is achieved (SA Health 2020).

Fujita et al. (2006) noted a case of occupational bronchial asthma and contact dermatitis reportedly caused by OPA exposure in a medical worker. The worker was a nurse in an endoscopy unit, with measured air concentrations of OPA throughout the unit ranging from 0.4 to 2.0 ppb (30 min sample). The unit had previously used glutaraldehyde solution to disinfect equipment, however, no respiratory or cutaneous symptoms were reported while handling glutaraldehyde. No details were provided on exposure controls in use. Owing to its low volatility, it is presumed that the skin may be a significant route of exposure (Anderson and Meada 2014). Other reported health effects from OPA exposure include eye and respiratory irritation, anaphylaxis, bronchial asthma, contact dermatitis, respiratory sensitization (mostly medical workers), and staining of unprotected skin (grey) (Streckenbach and Alston 2003; Sokol 2004; Cooper et al. 2008; Atiyeah et al. 2015; Henn et al. 2015). The National Institute for Occupational Safety and Health (NIOSH) undertook a health hazard evaluation of OPA in 8 healthcare facilities (Chen et al. 2015) and documented regular exposure to OPA in a variety of healthcare settings but reported skin and respiratory symptoms were rare.

Occupational hygienists rely on occupational exposure limits to assess hazardous levels of exposure to physical, chemical, and biological agents in the workplace and to verify that exposure control measures are adequate to prevent adverse health effects. In 2019, the American Conference of Governmental Industrial Hygienists (ACGIH) adopted a Threshold Limit Value–Surface Limit (TLV–SL) for OPA of 25 μg/100 cm^2^ (Naumann and Arnold 2019). The basis for the TLV–SL for OPA was to prevent the induction of dermal and respiratory sensitization following dermal exposure. TLV–SL are intended to supplement airborne TLVs to provide quantitative criteria for skin exposure, an important secondary route of exposure, at an acceptable surface concentration. Naumann and Arnold (2019) illustrate how the surface limit was derived using the murine local lymph node assay (LLNA) and effective concentration (EC3) value for OPA, with an adjustment factor applied for being a potent sensitizer. OPA has also been assigned a TLV–Ceiling airborne concentration of 0.1 ppb, measured as vapour fraction, to reduce the risk of developing skin, respiratory, and eye symptoms as well as sensitization.

NIOSH has undertaken the majority of previous work in health hazard evaluations and analytical method development for OPA in the air (and to some extent on surfaces) (Tucker 2008, 2014; Chen et al. 2015). Tucker (2008) proposed a potential surface measurement method consisting of Ghost Wipes dampened with dimethyl sulfoxide and water and analysed for degree of fluorescence using a portable fluorometer. This method was shown to be very sensitive, however, Chen et al. (2015) identified methodological issues when they collected 38 surface wipe samples from 8 facilities and found that the fluorometer method was only able to provide qualitative results (positive or negative presence) due to calibration curve drift over time. Tucker (2008) identified a less sensitive but field-based visual colorimetric surface wipe method, however, this provided only semiquantitative results not compatible for comparison with a TLV–SL. Yamamoto et al. (2020) recently described a very sensitive analytical method for the determination of OPA in air at the TLV–Ceiling threshold using LC–MS. To the best of our knowledge, there is no quantitative method for the determination of OPA surface contamination previously reported.

Sampling and analysis of aldehydes often involve derivatization prior to analysis, for both added stability and detection. Previously reported derivatizing agents include 2,4-dinitrophenylhydrazine (2,4-DNPH), 2,4,6-trichloro-phenylohydrazine (TCPH), cysteamine (2-aminoethanethiol), *O*-(2,3,4,5,6-pentafluorobenzyl) hydroxylamine (PFBHA), and morpholine (Osório and Cardeal 2013). The most common of which is 2,4-DNPH, which is what has been utilised to date for sample collection and analysis of OPA (Uchiyama et al. 2006; Tucker 2008, 2014; Chen et al. 2015; Yamamoto et al. 2020). While derivatization is an important part of the sampling it may not be suitable for surface sampling. Air sampling procedures use a DNPH-impregnated sampling medium to collect the analyte, however, if this was replicated for surface sampling it would likely leave yellow staining from the DNPH on the work surface.

The aim of this project was to address an existing gap by developing a quantitative sampling and analytical method for the determination of OPA work surface contamination. Such a method can be implemented into standard workplace risk assessment processes to assess against the ACGIH TLV–SL of 25 μg/100 cm^2^ for OPA. The method was developed in accordance with Occupational Safety and Health Administration (OSHA) evaluation guidelines (OSHA, 2021) and utilises commercially available wipes for sampling and a direct detection analysis approach using LC–ToF–MS. The reported method is simple and robust and negates the need for OPA derivatization, a common requirement for an airborne sampling of aldehydes.

## Materials and methods

### Overall approach

A new quantitative method for the sampling and direct analysis of OPA from work surfaces was developed to facilitate the evaluation of workplace assessment against the ACGIH TLV–SL. The method was developed against criteria outlined in the OSHA Evaluation Guidelines for Surface Sampling Methods (OSHA, 2021). These guidelines provide a uniform and practical means for evaluating surface sampling methods with regard to sampling media, sampling techniques, and sample preparation for analysis. The developed method was then applied to a real-world scenario by performing a worksite evaluation in a local hospital endoscopy unit in order to demonstrate its suitability.

### Materials

Solid *o*-phthalaldehyde (OPA) ≥ 99% (HPLC), Benzaldehyde-d_6_, 98 atom % D, and Acetonitrile (HPLC grade) ≥ 99.9%, were purchased from Merck Pty. Ltd. (Australia).

Previous studies by Tucker (2008) have indicated the purity of OPA solid drastically decreases with frequent opening and closing of the vial, exposing it to air. Hence, only small quantity vials were purchased (250 mg to 1 g) and once a vial was opened it was made immediately into a stock solution (10 mg/mL in acetonitrile). An aliquot of the stock solution was further diluted into a working solution (1 mg/mL in acetonitrile). The working solution was used for a maximum of 1 month before being replaced. When not in use the stock and working solutions were lightly flushed with N_2_ before being sealed and stored at –20 °C.

Benzaldehyde-d_6_, in solution (2.5 mg/mL in acetonitrile) was used as the internal standard (IS) for LC-ToF–MS analysis.

Three wipes were initially assessed: (i) Livingstone LIV-WIPE alcohol wipes (70% isopropyl alcohol swab, 65 × 56 mm), (ii) Ghost Wipes (prewetted with DI water, cut to 5 × 5 cm) and (iii) Berkshire Durx 670 dry wipes (cut to 5 × 5 cm and wet with 250 µL acetonitrile prior to sampling).

### Recovery from surfaces

To ensure OPA could be adequately removed from sampling surfaces it was liquid-spiked onto five 10 × 10 cm stainless-steel surfaces and five 10 × 10 cm glass surfaces, at the target concentration of 25 µg/100 cm^2^ (TLV–SL) (in 50 µL acetonitrile). This concentration was assessed as recommended in the OSHA evaluation guidelines for surface sampling (OSHA, 2021). These guidelines suggest performing 6 replicates, however, in this instance, the guidelines were deviated from and a total of 10 replicates were performed over 2 surface types.

The OPA was liquid-spiked (50 µL) onto the surface by pipetting in a grid pattern, and droplets were allowed to evaporate as determined by observation (a maximum of 30 s), and then promptly sampled. The area was sampled by applying firm hand pressure to the sampling medium and wiping across the surface (using a 10 × 10 cm template as a guide), starting at the top corner and wiping side to side while moving downward. The medium was folded with the contaminant side inward and repeated, wiping up and down while moving from one side to the other. The medium was folded again with the contaminant side inward and placed into an appropriately labelled 10 mL vial that had been wrapped in aluminium foil to reduce light exposure, ready for extraction and analysis via LC-ToF–MS.

The removal efficiency (% recovery) was deemed to be adequate by OSHA—as reported in the *Evaluation Guidelines for Surface Sampling M*e*thods* document (OSHA, 2021) if ≥50% of the contaminant that was placed on the surface was removed and accounted for by analysis. The recovery (%) of OPA was calculated by comparing the peak area of the surface spiked sample as a percentage of the peak area of standards made up to the same concentration (i.e. 25 µg/sample in 4 mL acetonitrile).

### Recovery from sampling medium

To ensure OPA could be adequately recovered from the sampling media it was liquid-spiked directly onto Livingstone alcohol wipes at 3.7 µg (LOQ), 25 µg (TLV–SL), 50 µg (2× TLV–SL) and 250 µg (10× TLV–SL), for 4 replicates. These concentrations and replicate numbers were selected as per the recommendations in the OSHA surface sampling evaluation guidelines (OSHA, 2021) which suggests assessing concentrations that represent the LOQ, 0.1, 1, and 10× the target concentration (the TLV–SL). An additional concentration point of 2× the target concentration was also assessed. Recovery of OPA spiked onto the wipe at 2.5 µg (0.1× TLV–SL) was also assessed, but the results are not presented here as this was below the determined limit of quantification.

After being loaded with OPA, the wipe was folded with the contaminant side inward and placed into an appropriately labelled 10 mL vial that had been wrapped in aluminium foil to reduce light exposure, ready for extraction and analysis vial LC-ToF–MS.

The extraction efficiency (% recovery) was deemed to be adequate if ≥75% of the contaminant that was placed on the wipe was extracted and accounted for by analysis (OSHA, 2021). The recovery (%) of OPA was calculated by comparing the peak area of the spiked sample as a percentage of the peak area of standards made up of the same concentration.

### Stability of OPA

To establish the stability of OPA under various conditions and ensure the analyte was stable over the required sampling and analysis timeframe a series of studies were conducted as outlined below.

#### In solution.

The stability of OPA in solution (i.e. the stock solution of OPA in acetonitrile and extracted samples) was monitored over a 31 day period. Three Livingstone LIV-WIPE were liquid-spiked at the target concentration (25 µg/100 cm^2^) and extracted and prepared for analysis via the method above. The samples were reanalysed via LC-ToF–MS an additional 5× over a 37 h period, then again on days 5, 10, 19, 24, and 31 following extraction. Fresh neat standards were also made up (from the original stock solution) on the day of each analysis to calculate recovery. To ensure there was no loss in the purity of the OPA samples the stability of the stock solution was also assessed by comparing the response (peak area) from each of these neat standards made up over the 31 days to the response (peak area) of the original standards. All samples were stored in the freezer at –20 °C, and thawed to room temperature prior to analysis.

#### On sampling media.

The stability of OPA on the sampling medium was assessed by spiking 33 Livingstone wipes with the target concentration of OPA (25 µg/100 cm^2^). The wipes were folded with the analyte inward and sealed in 10 mL vials wrapped in aluminium foil to reduce light exposure. Three samples were extracted and analysed on the day prepared. Fifteen samples were stored at a reduced temperature (4 °C) and the other 15 were stored at ambient temperature (22 °C ± 2), away from light. On days 5, 10, 17, 24, and 31, three samples were selected from each of the 2 storage sets, extracted and analysed. These concentrations and analysis time intervals were selected based on the OSHA surface sampling evaluation guidelines which suggest analysing 3 samples from each set approximately every fifth day, until days 15 to 18 (OSHA, 2021). Fresh neat standards were also made up on the day of each analysis to calculate recovery (%). It is recommended the recovery must remain above 75 % during storage (OSHA, 2021). When these conditions are not met, time requirements for when an analysis is completed should be implemented.

#### On surface.

The stability of OPA on a surface was investigated by liquid spiking OPA onto twelve 10 × 10 cm glass surfaces at the target concentration (25 µg/100 cm^2^). Three of the spiked surfaces were sampled immediately after the spike was observed to have dried (maximum 30 s) using Livingstone alcohol wipes. The remaining were sampled at 15 min intervals, in sets of 3.

### Sample extraction

To extract the wipe samples and prepare for analysis, 4 mL acetonitrile was added to each vial containing a wipe, and the vial was recapped. The vial caps were only removed when necessary and replaced as soon as possible to reduce air exposure, and the vials remained completely covered in aluminium foil during the extraction process to reduce exposure to light. 25 µL of internal standard solution (benzaldehyde-d_6_, 2.5 mg/mL in acetonitrile) was added to each sample. Each vial was loaded onto a Rotary Tube Mixer (Ratek Instruments) and rotated at low speed for 60 min. 1 mL aliquots were collected from each sample and pipetted into 2 mL crimp-cap LC vials for analysis via LC-ToF–MS.

### OPA quantification

A quantitative analysis method for OPA was developed using an Agilent Technologies 1260 Infinity LC system coupled to a 6,230 time-of-flight mass spectrometer (TOF) (Agilent Technologies Australia, Mulgrave, Victoria, Australia). Chromatographic separation was performed on a Phenomenex Kinetex 5 µm *C*_18_ 100 Å column (150 × 4.6 mm) fitted with a 4.6 mm *C*_18_ Phenomenex Security Guard cartridge (Phenomenex Australia, Lane Cove West, New South Wales, Australia). The column thermostat was maintained at 25 °C. The injection volume was 10 µL. An elution gradient using 0.1% v/v aqueous formic acid (mobile phase *A*) and 0.1% v/v formic acid in acetonitrile (*B*) was used, at a flow rate of 350 µL/min). The gradient was programmed as follows: 0 to 0.5 min at 90% *A*, linear decrease in *A* from 0.5 to 7.5 min to 50%, linear decrease in *A* from 7.5 to 10 min to 5%, linear decrease in *A* from 10 to 15 min to 0% and held at 0% *A* for 1 min. Total run time was 16 min per sample (with a 7 min post run equilibration time).

The mass spectrometer was operated with a dual nebulizer electrospray source in positive polarity, using the following settings for the duration of the run: drying gas (N_2_, 325 °C, 8 L/min) nebulizer pressure (35 psi), capillary voltage (3.5 kV), and fragmentor (175 V). The exact masses of the ion used to detect OPA and the internal standard were: OPA, *M* = 134.134 g/mol, monitored ion = 135.0440; Benzaldehyde-d_6_ (internal standard), *M* = 112.16 g/mol, monitored ion = 113.0870. Internal reference masses 922.0098 and 121.0509 *m/z* were monitored. The instrument resolution was set to high-resolution mode (vendor specifications of 22 000 FWHM measured at *m/z* 1,522). The mass range acquired was 100 to – 1,700 m/z.

Data were acquired using Agilent MassHunter Acquisition software (version 10.1) and analysed using MassHunter Qualitative (version 10.0) and Quantitative Analysis (version 10.2).

### Limit of detection and quantitation

The limit of detection (LOD) and limit of quantitation (LOQ) are measured as mass per sample. The values for these were 1.1 µg/sample (LOD) and 3.7 µg/sample (LOQ). LOQ was calculated using regression line data via the formula: *L*_RQ_ = 10*S*_YX_/*A*, where *L*_RQ_ is the reliable quantitation limit, *S*_YX_ is the SE of estimate for the regression line, and *A* is the analytical sensitivity (slope). Values of 2,489.9 and 915.9 were obtained for the slope and SE of estimate, respectively. Livingstone alcohol wipes were spiked with 0.7, 1.4, 2.1, 2.8, 3.5, 7, 10.5, 14, and 17.5 µg/wipe. These spiked wipes, and a sample blank were analysed with LC–MS, and data obtained was used to calculate the required parameters (SE of estimate and the slope) for the calculations as per OSHA recommendations (OSHA, 2021).

### Infield evaluation of OPA surface contamination

The objective of this part of the research was to perform a field evaluation of the analytical method and surface sampling procedure in actual representative workplace conditions in a local healthcare setting. The goal was to provide confidence that the sampling procedure was fit for purpose, analytically rigorous and adequately ruggedized. The surface sampling method was trialled for an assessment at a Pulmonary Function facility in a public hospital in South Australia, where an OPA-containing solution is used to sterilize equipment. Sampling occurred on a scheduled sterilization day and was indicative of an average workday in terms of the number of employees present, the quantity of OPA solution handled and amount of potential exposure to OPA.

The research project was assessed by the Human Research Ethics Committee, Office of Research Ethics, Compliance and Integrity, The University of Adelaide (Reference No. 35312). Additional approval was granted from the individual hospital to attend and sample.

Surface samples were taken using Livingstone LIV-WIPE alcohol wipes (prewetted with 70% isopropyl alcohol), in areas of high touch, such as the computer mouse, work benches and container handles. Where possible a 10 × 10 cm surface area was sampled (100 cm^2^), using a disposable template. Once wipe samples were collected, each wipe was placed in an individual vial, that had been wrapped in aluminium foil to reduce light exposure, and sealed and transported on ice to the laboratory for extraction and analysis. Gloves were replaced after every sample collection to reduce contamination.

OPA was extracted from the wipe into acetonitrile and analysed via LC-ToF–MS. To calculate the recovered mass (µg) of OPA, a calibration curve was produced by liquid spiking known amounts of OPA onto the wiping medium using concentrations ranging from 1 (LOD) to 250 µg/wipe (10× TLV–SL) and extracting using the procedure above. A blank wipe (no spike) was also included. The concentration of the unknown was calculated from the calibration curve.

All results are reported as µg/100 cm^2^. In the case where a 10 × 10 cm surface sample area was not possible (i.e. on irregular surfaces such as the handles of a container or bottle), the surface area was calculated and the concentration of OPA was reported as µg/100 cm^2^ using the following equation:

*M* (µg/100 cm^2^) = 100 × (Ms/*S*)

Where Ms = mass on the sampled surface (μg), and *S* = surface area sampled (cm^2^)

## Results and discussion

The proposed approach for quantification of *ortho*-phthalaldehyde contamination on work surfaces was developed through a comparison of different wipe types and extraction methods. The sampling method was intended to be easy and convenient, with an analytical approach that requires minimal treatment of the sampling medium prior to sampling or analysis. The 3 wipes that were assessed were: (i) Livingstone LIV-WIPE alcohol wipes (70% isopropyl alcohol swab, 65 × 56 mm), (ii) Ghost Wipes (prewetted with DI water, cut to 5 × 5 cm) and (iii) Berkshire Durx 670 dry wipes (cut to 5 × 5 cm and wet with 250 µL Acetonitrile prior to sampling). Of the 3 wipes assessed, Livingstone LIV-WIPE alcohol wipes (70% isopropyl alcohol swab, 65 × 56 mm) were ultimately selected as they provided the best recovery, and did not require additional treatment, i.e. cutting to size or addition of wetting agent, prior to sampling. Recovery of OPA from the other types of wipes tested is provided in Supplementary material. LC-ToF–MS was selected for analysis, over the traditionally used HPLC–UV, to remove the need for derivatization for the detection of OPA.

The recovery of OPA from stainless steel and glass surfaces using the Livingstone alcohol wipes was 71 % (Table 1), and the recovery of OPA from spiked sampling wipes was 89% (Table 2). The recovery from the stainless steel and glass surface using the Livingstone alcohol wipes (presented in Table 1) shows the recovery is above the recommended 50% recovery for every replicate. However, replicate 3 for the stainless steel is an outlier, at 91% recovery, while the other samples for the stainless-steel surface have a 60% to 70% recovery. It is hypothesized that the pressure placed on the wipe while sampling or the speed at which the wipe was moved across the surface may have varied compared to the other replicates, resulting in a higher recovery.

**Table 1.** Sampler removal efficiency data (% recovery) for OPA on glass and stainless-steel surfaces (*n* = 5).

Surface	Recovery (%)^a^					
	1	2	3	4	5	Mean (±SD)
Stainless steel	60.2	70.0	91.0	65.7	61.5	**69.7 (±11)**
Glass	79.6	67.1	66.0	80.9	64.8	**71.7 (±7.0)**
Total recovery						**70.7 (±9.4)**

^a^OSHA target recovery for spiked surfaces is >50%.

**Table 2.** Extraction efficiency (% recovery) of OPA from spiked Livingstone LIV-WIPE alcohol wipes (prewetted with 70% isopropyl alcohol) (*n* = 4).

Level		Recovery (%) ^a^				
Guidance target concentration	µg/sample	1	2	3	4	Mean (±SD)
LOQ (0.148 × TLV–SL)	3.7	81.8	85.6	100	>100 ^b^	89.1 (±7.8)
TLV–SL (target)	25	99.1	95.2	84.9	82.4	90.4 (±6.9)
2 × TLV–SL	50	81.3	88.4	95.8	76.6	85.5 (±7.3)
10 × TLV–SL	250	75.0	81.1	75.5	89.6	80.3 (±5.9)
Total recovery						86.2 (±8.0)

^a^OSHA target recovery from spiked sampling media is >75% but >90% preferred.

^b^This value was greater than 100% and has been omitted from the mean recovery

While this method of surface sampling meets the recommendations set out by OSHA (>50% recovery from spiked surfaces, and >75% recovery from spiked sampling media) (OSHA, 2021), other reported methods for surface sampling have demonstrated higher recoveries for OPA from glass surfaces (of up to 90%). Tucker (2008) used Ghost Wipes, cut to 2 × 10 cm, dried and wet with a dimethyl sulphoxide:water (40:60 v/v) mix prior to sampling. Three wipes were used to sample the same spot and combined for extraction with 12 mL acetonitrile, resulting in the recovery of 83% to 93%. In this instance, a smaller extraction volume was selected for increased sensitivity to suit analytical instrument capabilities. Cutting wipes to a smaller size was also considered to improve the recovery, without needing to increase the extraction solvent volume. However, the major benefits of the Livingstone alcohol wipe are that it is prewetted and individually wrapped, making it easy to send to workplaces with sampling templates, instructions, and collection vials, enabling occupational hygienists to collect the samples and return them to the laboratory with ease. The disadvantage of using a prewetted wipe is the inability to control the amount of wetting agent present on the wipe during the sampling or extraction, which may impact reproducibility. However, one of the key aims of this research was to develop an easy, convenient sampling method that required minimal treatment of the wipe prior to sampling or analysis. Using the Livingstone LIV-WIPE removes the requirement for any pretreatment of the wipe and the need to take additional solvents (such as dimethyl sulphoxide) to the site for sampling.

The LOD and the LOQ for this method were 1.1 and 3.7 µg/sample, respectively. Previous studies have reported more sensitive methods of detecting OPA than those reported here. For example, Yamamoto (2020) reported a LOQ of 0.165 µg/sample for using HPLC with tandem mass spectrometry (tripe quadrupole) analysing OPA derivatives. Tucker (2014) reported LODs of 0.016 µg/sample and 0.024 µg/sample using HPLC–UV and fluorescence analysis, respectively. However, these 3 methods were used to detect OPA in aerosols and vapours in air, so higher sensitivity is required. Tucker (2008) investigated surface wipe sampling with analysis of OPA (OPA-bis(DNPH)) via fluorescence measurement and visual colorimetric detection resulting in LODs of 0.2 µg/sample and 48 µg/sample, respectively. Nevertheless, our direct LC–TOF method offers significant advantages in terms of ease-of analysis and provides a foundation for further development of MS-based methods such as multiple reaction monitoring approaches that would address any need for greater sensitivity.

We sought to quantitate at ≤0.1× TLV–SL (2.5 µg/sample) in alignment with the OSHA guidance (OSHA, 2021), but this metric was not able to be met, and quantitation could only be performed to 0.148 × TLV–SL (3.7 µg/sample). This may be improved with more sophisticated instrumentation, but the trade-off may be that this can be expensive, and may not be available to some analytical laboratories. As a stand-alone analyser, the TOF platform offers advantages in terms of sensitivity (often 5 to 10 fold), dynamic range, speed of analysis and mass accuracy. This often makes it a platform of choice, particularly for analysis of compounds in the presence of complex matrices where chromatographically unresolved peaks are possible. Nevertheless, it is likely that similarly suitable methods could be developed on commonly employed triple-quadrupole platforms, utilising product ion monitoring to address requirements of sensitivity and accuracy. This method is still able to determine if a surface is contaminated with levels of OPA relative to the TLV–SL and can detect as low as 0.044× TLV–SL and quantitate levels of 0.148× TLV–SL. This method is sensitive enough to determine whether workers are at risk of dermal exposure to OPA from surface contamination at the TLV–SL.

### OPA stability

After 15 min of OPA on a surface being exposed to air and light, OPA could only be detected in one of the 3 samples, with a recovery of 6%. By 30 min there were no detectable levels of OPA on any of the surfaces. This indicates that the risk of dermal exposure to OPA from contaminated surfaces only persists for a short period which has practical implications for workers and workplaces. For example, the introduction of a 30 min ‘clearance window’ after cleaning up in rooms used to handle OPA, could significantly reduce the risk of dermal exposure to OPA arising from surface contamination. However, this short persistence is problematic for surface sampling methods and suggests if sampling is delayed too long after the handling of OPA it may not be detected. Because of this, it is recommended that surface wipe sampling is undertaken as close as practical to when the highest risk of dermal exposure to OPA would occur (i.e. directly after OPA is handled and used). These results are consistent with the work by Tucker (2008), where a notable drop in recovery was detected after 20 to 50 min of OPA being exposed to air.

The assessment of storage stability (presented in Table 3) shows OPA remained stable (>75% recovery) on the wipe for at least 10 days in refrigerated storage (4 °C), but less than 5 days when stored under ambient conditions. Hence, it is recommended that samples in transit should be kept in a portable cooler, and where possible, transported with ice packs to keep the samples cool. Once in the laboratory, samples should be extracted on the same day as they arrive or stored in the refrigerator (4 °C). Stock solution and extracted samples remained stable for 31 days at –20 °C. Therefore, it is recommended that extracts should be stored in the freezer (–20 °C) if they are unable to be analysed on the same day as the extraction.

**Table 3.** Storage test for OPA on a Livingstone LIV-WIPE (70% isopropyl alcohol swab, 65 × 56 mm) (*n* = 3).

Time (days)	Ambient storage Recovery %			Refrigerated storage Recovery %		
0	99.1	95.2	84.9	99.1	95.2	84.9
5	66.5	89.5	75.9	99.7	87.7	75.7
10	68.8	97.8	62.9	91.5	84.7	95.3
17	68.1	65.5	62.0	70.7	69.9	76.6
24	72.9	69.9	71.6	83.4	69.5	81.0
31	67.2	64.9	65.5	70.7	65.2	68.0

### Infield evaluation of OPA surface contamination

The applicability of the developed sampling and analysis method was demonstrated via an infield assessment at a hospital sterilising unit. The results of this assessment showed 12 of the 14 samples collected had OPA concentrations below the limit of quantitation (Table 4). The main location of OPA surface contamination was on the sterilizing container, with both samples exceeding recommended TLV–SL of 25 μg/100 cm^2^ (the handle of the container having 56 µg/100 cm^2^, and the front face of the jug having 28 μg/100 cm^2^). This emphasises the need for workers to wear appropriate personal protective equipment (PPE) including fluid-resistant gowns, nitrile gloves (or double latex gloves), and eye protection (goggles or face shields), to reduce the likelihood of direct skin contact. Workers should also use correct PPE when handling equipment associated with the OPA cleaning process, even if OPA is not in use at the time. This is because equipment previously cleaned with the OPA also had low levels of OPA surface residue. However, these levels were below the limit of quantitation.

**Table 4.** Concentrations of OPA were detected on surfaces within the Pulmonary Function facility in a large public hospital in South Australia, Australia.

Sample description	OPA concentration (µg/100cm^2^)^ab^
LEFT side of OPA processing area (benchtop)—3 samples taken in the same area (no overlapping of sampling areas).	N/D
	N/D
	N/D
RIGHT side of OPA processing area (benchtop)—3 samples taken in the same area (no overlapping of sampling areas).	N/D
	N/D
	N/D
Front edge/lip of OPA processing bench—53 × 3 cm sampling area, a single sample taken	N/D
Outside surface of the sterilizing container (front)	**27.9**
Outside surface of the sterilizing container (handle—~88 cm^2^ surface area)	**55.5**
Sink (next to drain where neutralized Cidex solution is disposed)	N/D
Cidex bottle (side)	N/D
Cidex bottle (handle—13 × 3.5 × 2 cm)	N/D
Attachment to the mouthpiece of spirometer and diffusion analyser (pneumotech flow sensor)—after being cleaned/processed with Cidex solution	**Detected** **<LOQ** ^b^
Mouse of computer	N/D
On-site blank (control sample)	N/D

^a^N/D = OPA was not detected, or below the limit of detection (1.1 µg/100 cm^2^)

^b^Detected <LOQ = OPA was detected in the sample but was below the limit of reliable quantitation (3.7 µg/100 cm^2^)

As previously mentioned, Chen et al. (2015) and Tucker (2008) applied surface wipe techniques for OPA exposure measurement as part of a site evaluation. However, the reported method was only able to provide qualitative results (positive or negative presence). By comparison, the method proposed in this paper is capable of quantitative measurements for assessment against the TLV–SL.

This developed approach for the determination of OPA on work surfaces is intended to supplement airborne measurements and provide a quantitative exposure assessment tool for exposure via contaminated surfaces. Further optimisation of MS detection strategy and mobile phase composition, taking into consideration the stability of the analyte, would be required for a highly sensitive, validated method translatable to commercial analytical settings. However, this method demonstrates it is possible to meet the needs of both occupational hygienists and analytical chemists with a simple, rapid sampling, and quantification strategy that does not require derivatization or wipe impregnation. It may be useful when incorporated into workplace risk assessment procedures as part of a comprehensive exposure control program. When used in conjunction with a thorough occupational hygiene program that includes hazard communication, engineering controls, and personal protective equipment, skin exposure and consequent sensitization risks in the workplace can be minimized.

## Conclusion

This research has demonstrated the successful development of an appropriate approach for the quantitative evaluation of OPA surface contamination. It is capable of determining OPA on glass and stainless-steel surfaces below the TLV–SL concentration. The reported method uses readily available commercial wipes (Livingstone LIV-WIPE alcohol wipes; 70% isopropyl alcohol, 65 × 56 mm) and a direct-detection analytical approach (LC-ToF–MS) without the need for derivatization typically required for airborne sampling of aldehydes. It was applied to an infield surface assessment of a hospital sterilization unit and detected OPA above the TLV–SL on sterilising containers. This method has the potential to be incorporated into workplace risk assessment processes to ensure workers are not dermally exposed to OPA via contaminated surfaces in high-risk workplaces. OPA remained stable (>75% recovery) on the wipe for at least 10 days in refrigerated storage (4 °C), therefore it is recommended that samples in transit should be kept in a portable cooler, and where possible, transported with ice packs to keep the samples cool.

## Supplementary Material

wxad039_suppl_Supplementary_Materials

## Data Availability

The authors confirm that the data supporting the findings of this study are available within the article [and/or] its supplementary materials.

## Declaration

This research was supported with funds by WorkSafeBC, The Workers’ Compensation Board of British Columbia. The views, findings, opinions, and conclusions expressed herein do not represent the views of WorkSafeBC.
