# Supplementary material for: An approach to quantify ortho-phthalaldehyde contamination on work surfaces
Source: Ann Work Expo Health. 2023 Jul 12;67(7):886–94. doi: 10.1093/annweh/wxad039 (PMC10410490; doi:10.1093/annweh/wxad039)
Supplement: wxad039_suppl_Supplementary_Materials [file wxad039_suppl_supplementary_materials.docx]

**An approach to quantify *ortho*-phthalaldehyde contamination on work surfaces**

**Keywords:** *o*-phthalaldehyde, surface sampling, Threshold Limit Value-Surface Limit (TLV-SL)

Word count: 4887

Caitlyn A. Rogers^1^, Sharyn E. Gaskin^1*^, Leigh D. Thredgold^1^, Tara L. Pukala^2^

^1^Adelaide Exposure Science and Health, School of Public Health, University of Adelaide, Adelaide, South Australia, Australia 5005; ^2^Department of Chemistry, School of Physical Sciences, University of Adelaide, Adelaide, South Australia, Australia 5005

*corresponding author: Sharyn Gaskin, 28 Anderson St, Thebarton SA 5031, [Sharyn.gaskin@adelaide.edu.au](mailto:Sharyn.gaskin@adelaide.edu.au) , T: (+61) 08 8313 4958, F: (+61) 08 8313 4955.

Author contributions: S.G. principal investigator, study design, organised field study and obtained ethics approval, led the manuscript writing; C.R. data acquisition, analysis and interpretation, and assisted with the manuscript writing; L. T. co-investigator, assisted with laboratory set up, assisted with ethics application, assisted with writing; T. P. co-investigator, instrument maintenance, assisted with data interpretation and analysis, assisted with writing.

**Keywords:** *o*-phthalaldehyde, surface sampling, Threshold Limit Value-Surface Limit (TLV-SL)

**Supplementary material**

Removal efficiency of OPA from the stainless-steel surface using the three sampling media is presented in Table S1.

While all the wipe types had an average recovery >50 %, the LIV-WIPE® performed better with an overall recovery of 76 %. In addition, the LIV-WIPE® demonstrated an average recovery >50 % at all three test concentrations, whereas the Ghost™ Wipes did not meet this criteria. Compared to the Durx® 670, the Ghost™ Wipes had higher recoveries, but also had more variability between samples.

The Livingstone LIV-WIPE® has additional advantages over the other two wipes. The LIV-WIPE® comes pre-wetted and individually wrapped, already at the ideal size. The LIV-WIPE® comes cut to 65 x 56 mm, making it large enough to perform effective sampling, while also being small enough to fit in standard extraction tubes, and perform extraction without needing to increase the volume of extraction solvent used. In contrast, the Durx® 670 and the Ghost™ Wipes were too big to be used in the form supplied and needed to be cut down to a similar size to the LIV-WIPE®. This increases the chance of contamination when preparing the Durx® 670 and the Ghost™ Wipes for sampling. The Durx® 670 also needs to be manually wetted before sampling, adding additional complications in terms of transporting samples to and from the laboratory.

The Livingstone LIV-WIPE® is the most convenient choice as it does not need any pre-treatment prior to sampling, performs the best in terms of recovery and is cost effective and readily available.

Table S1. Removal efficiency (% recovery) of OPA from stainless-steel surfaces using 3 different sampling media (n=3)

|  | Surface Concentration (µg/100cm^2^) | Recovery (%) | | | Average Recovery  % (±SD) |
| --- | --- | --- | --- | --- | --- |
| Livingstone LIV-WIPE® (70% isopropyl alcohol) | 2.5 | 58.4 | 50.8 | 56.1 | 55.1 (±3.2) |
|  | 25 | 105 | 83.4 | 97.4 | 95.1 (±8.8) |
|  | 50 | 77.0 | 72.7 | 88.2 | 79.1 (±6.5) |
|  | **Overall** |  |  |  | **76.5 (±17)** |
| Ghost™ Wipes (D.I water) | 2.5 | 59.2 | 28.9 | 44.3 | 44.1 (±12) |
|  | 25 | 79.7 | 66.6 | 74.4 | 73.6 (±5.4) |
|  | 50 | 74.2 | 56.6 | 58.5 | 63.1 (±7.9) |
|  | **Overall** |  |  |  | **60.3 (±15)** |
| Burkshire Durx® 670 dry wipe, manually wetted with acetonitrile | 2.5 | 64.2 | 64.9 | 52.7 | 60.6 (±5.6) |
|  | 25 | 61.8 | 55.8 | 45.6 | 54.1 (±6.3) |
|  | 50 | 74.0 | 65.1 | 67.9 | 69.0 (±3.7) |
|  | **Overall** |  |  |  | **61.2 (±8.1)** |
